# Characterization of Natural Killer Cell Phenotype and Function during Recurrent Human HSV-2 Infection

**DOI:** 10.1371/journal.pone.0027664

**Published:** 2011-11-15

**Authors:** Niklas K. Björkström, Alexandra Svensson, Karl-Johan Malmberg, Kristina Eriksson, Hans-Gustaf Ljunggren

**Affiliations:** 1 Center for Infectious Medicine, Department of Medicine, Karolinska Institutet, Karolinska University Hospital Huddinge, Stockholm, Sweden; 2 Department of Rheumatology and Inflammation Research, University of Gothenburg, Gothenburg, Sweden; Centre de Recherche Public de la Santé (CRP-Santé), Luxembourg

## Abstract

Human natural killer (NK) cell differentiation, characterized by a loss of NKG2A in parallel with the acquisition of NKG2C, KIRs, and CD57 is stimulated by a number of virus infections, including infection with human cytomegalovirus (CMV), hantavirus, chikungunya virus, and HIV-1. Here, we addressed if HSV-2 infection in a similar way drives NK cell differentiation towards an NKG2A^-^NKG2C^+^KIR^+^CD57^+^ phenotype. In contrast to infection with CMV, hantavirus, chikungunya virus, and HIV-1, recurrent HSV-2 infection did not yield an accumulation of highly differentiated NK cells in human peripheral blood. This outcome indicates that human HSV-2 infection has no significant imprinting effect on the human NK cell repertoire.

## Introduction

Genital herpes simplex virus type 2 (HSV-2) infection is the most common sexually transmitted ulcerative disease worldwide [1]. The clinical presentation of HSV-2 infection ranges from asymptomatic disease to recurrent and severe episodes of genital and non-genital infection. Furthermore, recurrent HSV-2 infection is a major risk factor for the acquisition, transmission, and progression of HIV-1 infection [1]. Adaptive cellular immunity has been shown to be important in clearing HSV-2 infection both in murine models and human studies [1]. Work from experimental models of genital HSV-2 infection suggests that natural killer (NK) cells may additionally be important in early control of infection [1]. Interestingly, HSV-2 has been reported to disseminate in humans during temporary episodes of NK cell deficiencies [1]. However, the impact of infection with HSV-2 on the NK cell repertoire in humans has not been studied.

NK cells provide a first line of defense against many virus infections [2]. In humans, the most convincing evidence for this comes from patients with selective NK cell deficiencies. These individuals often suffer from severe infections by viruses belonging to the herpesvirus family [3]. Interestingly, many human herpesviruses have also developed immune evasion mechanisms that specifically target NK cells [2]. In experimental models, NK cells can recognize, and have even been described to form memory against, cytomegalovirus (CMV) [4]. In humans, infection by a multitude of viruses, including CMV, hantavirus, chikungunya virus, and HIV-1 has been reported to cause a shift in the NK cell repertoire towards an accumulation of more terminally differentiated NK cells [5], [6], [7], [8], [9]. Such NK cell differentiation is characterized by a gradual shift from more immature NKG2A^+^CD62L^+^CD57^−^KIR^−^CD56^bright^ NK cells to NKG2A^+/−^CD62L^+/−^CD57^−/+^KIR^−/+^CD56^dim^ intermediates that progress further towards terminally differentiated NKG2A^−^CD62L^-^CD57^+^KIR^+^CD56^dim^ NK cells [10], [11], [12]. This NK cell differentiation is associated with multiple phenotypic and functional changes, including low expression of cytokine- and chemokine-receptors, a gradual decline in proliferative capacity and responsiveness to cytokines, and increased ability to perform cytotoxic responses [10], [11], [12].

In the current study, we examined the phenotype and function of NK cells and, furthermore, specifically addressed whether a HSV-2 infection could drive the human NK cell repertoire towards an accumulation of terminally differentiated NKG2A^-^NKG2C^+^KIR^+^CD57^+^ cells as has been observed in other human viral infections.

## Results

### Characterization of NK cell differentiation status during recurrent HSV-2 infection

**Figure 1:**
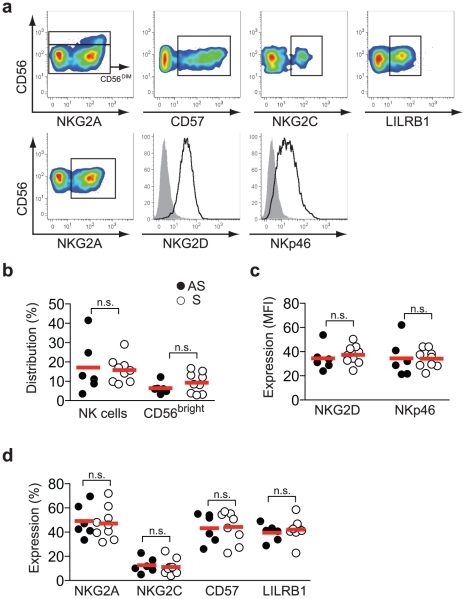
Characterization of NK cell differentiation in symptomatic HSV-2 infected patients. (A) Representative stainings for identification of CD56^bright^ NK cells, and NKG2A-, CD57-, NKG2C-, LILRB1-, NKp46-, and NKG2D-positive CD56^dim^ NK cells in PBMC from one patient with recurrent HSV-2 infection. For NKp46 and NKG2D, solid grey histograms display isotype controls and black lines display the respective specific stainings. (B) Frequency of NK cells out of total lymphocytes and CD56^bright^ NK cells out of total NK cells. (C) Expression levels (mean fluorescence intensity, MFI) of NKG2D and NKp46 on CD56^dim^ NK cells. (D) Expression (%) of NKG2A, NKG2C, CD57, and LILRB1 on CD56^dim^ NK cells. In (B) through (D), closed circles represent asymptomatic (AS) HSV-2 seropositive individuals (*n* = 6) and open circles represent patients with recurrent (S) HSV-2 infection (*n* = 9). In (B) through (D), the Mann-Whitney rank sum test was used, n.s., not significant, bars represent mean.

To characterize the phenotype and function of human NK cells, and to specifically study if HSV-2 infection drives human NK cell differentiation, peripheral blood was obtained from patients with recurrent genital herpes as well as from healthy, asymptomatic, HSV-2 seropositive individuals. On this material, we performed a detailed characterization of NK cells using multi-color flow cytometry, assessing the phenotype, functionality, and differentiation status of *ex vivo* isolated peripheral blood NK cells. The results allowed us to evaluate specifically if recurrent HSV-2 infections led to changes in the NK cell repertoire compared to a latent asymptomatic infection. Patients with recurrent HSV-2 infection and HSV-2 seropositive controls had equal numbers of total NK cells as well as equal proportions of CD56^bright^ NK cells out of total NK cells (Fig. 1A and 1B). Early and late differentiated NK cells can be identified by expression patterns of NKG2A and CD57 [10], [12]. More immature CD56^bright^ NK cells are uniformly NKG2A^+^CD57^−^, whereas CD56^dim^ NK cell differentiation is characterized by the gradual loss of NKG2A coupled to the acquisition of CD57 [10], [12]. In the present study groups, expression patterns of NKG2A and CD57 on CD56^dim^ NK cells from patients with recurrent HSV-2 infection were strikingly similar to those of NK cells from HSV-2 seropositive healthy individuals (Fig. 1A and 1D). Furthermore, inhibitory LILRB1 and activation receptors NKG2D and NKp46, which are also altered during NK cell differentiation [10], [11], [12], remained unaffected when comparing the two groups (Fig. 1A, 1C, and 1D). These findings contrast with those from patients with, e.g., HIV-1, chikungunya virus, or hantavirus infection in whom the infections drive the increased accumulation of highly differentiated NK cells [6], [7], [9].

From our investigation of NKG2C expression in patients with recurrent HSV-2 infection, on average 10% (range 4% to 25%) of their CD56^dim^ NK cells expressed the receptor (Fig. 1A and 1D). However, no specific expansion of NKG2C^+^ NK cells was detected in the patients as compared to the HSV-2 seropositive healthy individuals (Fig. 1D). The CMV serostatus was not available for either group, representing a possible limitation in the analysis of NKG2C expression. This issue remains unresolved, since previous work associated expression of the activation receptor NKG2C on NK cells with human CMV infection, and no other herpesvirus has, *per se*, been linked to enhanced NKG2C expression [5]. Furthermore, previous reports have shown that CMV serostatus affects the frequency of NKG2C^+^ NK cells in patients with HIV-1 [13]. Nevertheless, the presence of NKG2C^+^ NK cells in all subjects investigated in this study would argue for a high prevalence of CMV in both groups (Fig. 1D).

### The KIR repertoire of CD56^dim^ NK cells remain intact in recurrent HSV-2 infection

**Figure 2:**
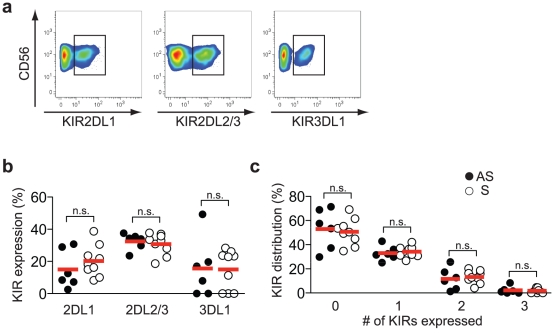
KIR expression on NK cells from symptomatic HSV-2 patients. (A) Representative stainings for KIR2DL1-, KIR2DL3-, and KIR3DL1-positive CD56^dim^ NK cells from one patient with recurrent HSV-2 infection. (B) KIR expression on CD56^dim^ NK cells. (C) Frequency of CD56^dim^ NK cells expressing 0, 1, 2, or 3 KIRs, as determined after a Boolean gating analysis for KIR2DL1, KIR2DL2/3, and KIR3DL1. In (B) and (C), closed circles represent asymptomatic (AS) HSV-2 seropositive individuals (*n* = 6) and open circles represent patients with recurrent (S) HSV-2 infection (*n* = 9). In (B) and (C), the Mann-Whitney rank sum test was used, n.s., not significant, bars represent means.

Inhibitory KIR (killer cell immunoglobulin-like receptor) expression on NK cells is primarily confined to the CD56^dim^ NK cell subset [10]. NK cell differentiation is associated with a sequential acquisition of KIRs [10], [11], [12]. Except for preserving tolerance and regulating NK cell recognition of target cells, NK cells must express at least one inhibitory KIR with a self-ligand present in the host to become educated [14]. Here, we investigated both the KIR specificity (i.e., expression of individual KIRs) and the KIR distribution pattern (i.e., the combination in which KIRs are expressed) by using three different anti-KIR antibodies for co-staining of CD56^dim^ NK cells from the patients with recurrent HSV-2 infection (Fig. 2A). NK cells from these patients showed a variegated KIR expression (Fig. 2B) with, amongst the KIR^+^ cells, a dominance of single KIR-expressing cells (Fig. 2C). This pattern was similar to the KIR profile of the HSV-2 seropositive healthy individuals (Fig. 2B and 2C). Furthermore, the two groups had on average 50% KIR-negative NK cells (Fig. 2C). These data provide additional evidence that no differentiated NK cells accumulated during recurrent HSV-2 infection, as they did in the other viral infections described above. For instance, this pattern contrasts with that of the KIR profile for the differentiated NKG2C^+^CD57^+^ NK cells that expand during acute hantavirus infection. In these hantavirus-infected individuals, the expanded NK cells had a profile that was skewed towards expression of a single inhibitory KIR [7].

### NK cell degranulation capacity is intact during recurrent HSV-2 infection

**Figure 3:**
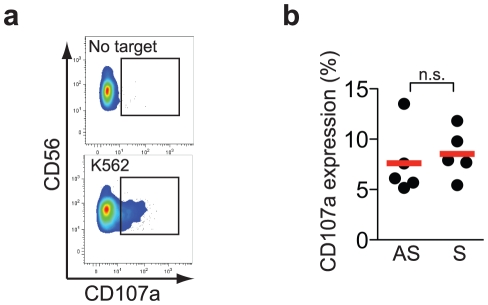
NK cells from patients with symptomatic HSV-2 infection retain their capacity to degranulate. (A) Representative staining for CD107a expression on unstimulated and K562 target cell-stimulated NK cells from one patient with symptomatic HSV-2 infection. (B) Level of CD56^dim^ NK cell degranulation against K562 cells for asymptomatic (AS) HSV-2 seropositive individuals (*n* = 5) and patients with recurrent (S) HSV-2 infection (*n* = 5). The Mann-Whitney rank sum test was used, n.s., not significant, bars represent means.

Finally, to address if a possible defect in NK cell degranulation might represent one factor behind recurrent HSV-2 infection, the capacity of resting unmanipulated NK cells to degranulate against K562 target cells after a six-hour co-incubation time was evaluated by assessment of CD107a expression (Fig. 3A). NK cells from patients with recurrent HSV-2 infection were equally efficient as NK cells from HSV-2 seropositive healthy individuals in upregulating CD107a (Fig. 3B). Thus, unlike the degranulation defects seen in some patient groups with familial hemophagocytic lymphohistiocytosis [15], NK cells from patients with recurrent HSV-2 infection seem to have an intact capacity to degranulate.

## Discussion

Several limitations of our study have to be considered. First, one explanation for the unaltered NK cell differentiation status found in peripheral blood of patients with recurrent HSV-2 infection might be that HSV-2 exerts only local effects in the genital mucosa. Support for this conclusion comes from studies of patients with celiac disease [16]. There, only intraepithelial CD8 T cells in the gut lumen, i.e., at the site of inflammation, became activated and expressed high levels of NKG2C, whereas the corresponding T cells in peripheral blood were unaffected [16]. Thus, we cannot exclude that tissue-resident NK cells are differentiating in response to HSV-2 infection. A characterization of genital mucosa-associated NK cells during recurrent HSV-2 infection might reveal a different pattern of NK cell differentiation.

Second, we did not have access to clinical data documenting when the patients with recurrent HSV-2 infection had their last episode of infection. It is possible that signs of NK cell differentiation occur only transiently in conjunction with active infection. However, during both acute hantavirus and acute chikungunya virus infections [7], [9], and during CMV reactivation [8], alterations in the NK cell repertoire are evident in peripheral blood months after infection.

Third, since HSV-2 in most individuals is a lifelong latent infection, the absence of a HSV-2 seronegative group of healthy individuals in the study design precluded our ability to test for NK cell differentiation changes that occurred solely as a consequence of HSV-2 serostatus. Instead, our study focused on determining the impact of recurrent HSV-2 infections, defined as six or more annual episodes of clinical disease, on the NK cell repertoire. Whereas changes in the NK cell repertoire attributed to HSV-2 serostatus, *per se*, have not been analyzed in detail before, it has been shown that neither HSV-1 nor Epstein-Barr virus serostatus has had any major impact on the expression of NKG2C; furthermore, CMV serostatus causes no alterations in KIR or LILRB1 expression [5].

In summary, this study of recurrent HSV-2 infection in humans revealed a distinct difference from the outcome of infection with human CMV, hantavirus, chikungunya virus, or HIV-1 [5], [6], [7], [9], all of which can drive NK cell differentiation towards the accumulation of terminal effector cells. Instead, the data suggest that HSV-2 infection, in contrast to other infections, leaves the NK cell repertoire unaltered.

## Materials and Methods

### Ethics statement

All included patients and healthy individuals gave written informed consent to participate in the study and the Ethics Committee of the University of Gothenburg, Sweden, granted permission for the study.

### Patient material

Blood samples were obtained from patients with recurrent HSV-2 infection were obtained. These patients had a typical history of recurrent genital herpes, with six or more annual relapses of infection. As controls, asymptomatic HSV-2 seropositive individuals were recruited during routine screenings. The latter HSV-2 seropositive healthy individuals were interviewed thoroughly before being classified as asymptomatic carriers. HSV-2 seropositivity was verified by ELISA in both groups. Peripheral blood mononuclear cells (PBMC) were isolated from whole fresh blood of both groups by centrifugation on Ficoll-Hypaque and were vitally frozen in 95% fetal calf serum and 5% DMSO in liquid nitrogen for later usage.

### ELISA for detection of HSV-2 specific antibodies

Plasma was screened for HSV-2 specific antibodies using ELISA as described [17].

### Phenotyping of NK cells by flow cytometry

Flow cytometry stainings were performed as previously described on thawed PBMC [18]. Briefly, Fc receptors were blocked using 0.5mg/ml of intravenous immunoglobulin for 20 min on ice. Next, ethidium monoazide bromide was added (1 µg/ml), and the cells were incubated 10 min on ice in the dark followed by a 10 min exposure to bright light on ice for later visualization of dead cells. Finally, cells were stained with saturating amounts of the desired monoclonal antibody (mAb) combination and incubated 20 min on ice in the dark. Data were acquired on a CyAn™ ADP LX 9-color flow cytometer and analyzed with FlowJo software version 9.3. Commercially available mAbs against the following proteins were used: NKG2A, KIR2DL1, KIR2DL2/3, KIR3DL1, CD57, NKG2C, LILRB1, NKp46, NKG2D, CD56, CD3, and CD14.

### Degranulation assay

For analysis of NK cell function, CD107a degranulation assays were performed as previously described [19]. Briefly, thawed PBMC that had been rested over night in complete medium at 37°C were cocultured with K562 target cells (American Type Culture Collection, Manassas, VA, USA) at a 10∶1 E:T ratio for six hours. A commercially available mAb against CD107a was added at the start of the assay. Monensin was added after one hour of coculture. After six hours, the samples were stained with mAbs against CD56, CD3, and CD14, and immediately acquired, as described above.

### Statistical analysis

Statistical analysis was performed using Graphpad software version 5.0, and the Mann-Whitney rank sum test was performed.
